# The Relative Efficacy and Safety of Monotherapies for Alopecia Areata: A Network Meta‐Analysis Study

**DOI:** 10.1111/jocd.70185

**Published:** 2025-04-15

**Authors:** Aditya K. Gupta, Mary A. Bamimore, Paradi Mirmirani, Vincent Piguet, Mesbah Talukder

**Affiliations:** ^1^ Mediprobe Research Inc. London Ontario Canada; ^2^ Division of Dermatology, Department of Medicine, Temerty Faculty of Medicine University of Toronto Toronto Ontario Canada; ^3^ Department of Dermatology The Permanente Medical Group Vallejo California USA; ^4^ Department of Dermatology University of California, San Francisco San Francisco California USA; ^5^ Department of Dermatology Case Western Reserve University Cleveland Ohio USA; ^6^ Division of Dermatology, Women's College Hospital Toronto Ontario Canada; ^7^ School of Pharmacy BRAC University Dhaka Bangladesh

**Keywords:** alopecia, androgenetic alopecia, autoimmune

## Abstract

**Background:**

Scant evidence exists for the relative efficacy of therapies for alopecia areata (AA)—including those approved by the Food and Drug Administration, namely, baricitinib, deuruxolitinib, and ritlecitinib.

**Aims:**

We determined the relative efficacy and safety of monotherapy with janus kinase inhibitors (JAKIs), apremilast, and dupilumab.

**Methods:**

Following a systematic review, we conducted Bayesian network meta‐analysis (NMAs) that produced Surface Under the Cumulative RAnking (SUCRA) values and point estimates for pairwise relative effects; we also performed sensitivity analyses.

**Results:**

In total, regimens with eight various JAKIs were compared, namely, ruxolitinib, ATI‐501, baricitinib, brepocitinib, deuruxolitinib, ivarmacitinib, ritlecitinib, and tofacitinib. Our analyses ranked “deuruxolitinib 12 mg twice daily for 24 weeks,” the most efficacious insofar as “proportion of participants achieving SALT ≤ 20 at 24 weeks” (SALT_20_) (SUCRA = 92.6%), and “proportion of participants achieving SALT ≤ 10 at 24 weeks” (SALT_10_) (SUCRA = 97.7%). As per SALT_20_, the highest‐ranked regimen was more efficacious than “baricitinib 2 mg once daily for 24 weeks” (odds ratio [OR] = 5.37, 95% credible interval [CI] = 1.59, 13.70, *p <* 0.05). Furthermore, the efficacy of the FDA‐approved JAKIs exhibited a dose‐dependent relationship; for instance, baricitinib 4 mg once daily for 24 weeks was more efficacious than “baricitinib 2 mg once daily for 24 weeks” in terms of SALT_20_ (OR = 2.25, 95% CI = 1.56, 3.21, *p* < 0.05). Results from our sensitivity analyses support that our base analyses were robust.

**Conclusions:**

We produced high‐quality evidence on the comparative effectiveness of monotherapies for AA with various regimens of 8 JAKIs, including the FDA‐approved ones. Our findings can improve clinicians' decision‐making and update guidelines for medical practice.

## 1 Introduction

Alopecia areata (AA), an immune‐mediated disorder, manifests as non‐scarring hair loss either in patches, across the whole scalp (“alopecia totalis”)—or the entire body (i.e., “alopecia universalis”) [1]. The hair loss in AA can have a significant psychosocial impact [2] and can raise concerns for other health conditions including atopic disorders [3] and other autoimmune conditions, for example, rheumatoid arthritis and autoimmune thyroiditis [4]. Inhibition of janus kinases, a family of enzymes linked to immunometabolism [5], has demonstrated therapeutic potential in numerous clinical trials of various immune disorders [6]. For example, the literature supports that inhibition of janus kinase 1 (JAK1) improves inflammatory bowel disease [7]; JAK1/3 inhibitors are a therapeutic modality for ankylosing spondylitis [5]. The United States Food and Drug Administration (FDA) has approved the following for AA: ritlecitinib 50 mg once daily, baricitinib 2 mg once daily, baricitinib 4 mg once daily, and deuruxolitinib 8 mg twice daily.

Multiple clinical trials have examined the impact of therapy with various JAK inhibitors (JAKIs) for AA [5, 8], albeit the effects of some have not been compared in head‐to‐head studies. A network meta‐analysis (NMA) is a statistical technique where numerous therapies' comparative effects can be simultaneously determined. Since the publication of previous NMA studies on AA therapies [5, 9], newer trial data have been published. Using the most updated literature, we simultaneously determined the relative efficacy and safety of monotherapy with dupilumab, apremilast, and JAKIs for AA.

## 2 Methods

The entire conduct of the present NMA study was consistent with the Preferred reporting items for systematic review and meta‐analysis (PRISMA) guidelines for NMAs. The protocol for this work was prospectively registered with the International Platform of Registered Systematic Review and Meta‐analysis Protocols (INPLASY) (ID: INPLASY202510120).

On January 7, 2025, we systematically searched the literature through PubMed and Scopus to identify studies whose data were analyzed in our NMAs. Management of the search process was aided by Rayyan [10] and the Systematic Review Accelerator (SRA) [11]. The systematic search, screenings, and full‐text review were conducted independently by two authors (MAB and AKG); any discordance was resolved through discussion with a third author (MT). Extracted data were organized into spreadsheets.

The outcomes we chose were those based on the Severity of Alopecia Tool (SALT) score, a standardized hair loss measurement tool whose values range between 0 and 100 (inclusive); a value of 100 translates to complete hair loss while 0 corresponds to no hair loss; hence, a decreasing score corresponds to increased hair growth (i.e., a more favorable outcome) [12].

Eligible studies were RCTs for AA that investigated the efficacy of monotherapies with JAKIs, dupilumab, and apremilast in terms of four outcomes, namely, (1) “percentage reduction in SALT score at 24 weeks from baseline,” (2) “proportion of participants achieving a SALT score of 20 (or less) at 24 weeks from baseline,” (3) “proportion of participants achieving a SALT score of 10 (or less) at 24 weeks from baseline,” and (4) “proportion of participants achieving at least a 90% relative reduction in SALT at 24 weeks from baseline. Included studies” evidence quality was evaluated using the Cochrane Collaboration's Risk of Bias (RoB) tool [13]. The four efficacy‐related endpoints were our primary outcomes; as a secondary outcome, we estimated regimens' relative safety as per “discontinuation due to adverse events (AEs) 24 weeks from baseline”. For sensitivity analyses, we conducted two separate network meta‐regressions, where one controlled for confounding due to variation in age and the other ecologically controlled for confounding due to variation in sex. The network meta‐regressions were ecological because unit of analyses was at the level of the study.

Network plots, which are graphs of nodes and edges, were used to depict direct evidence (i.e., modalities whose efficacy was compared in actual head‐to‐head trials). Transitivity was qualitatively assessed by reviewing study characteristics of included trials, and the geometry of the plots determined whether a node‐splitting of inconsistency could ensue.

For each network, we ran a Bayesian NMA with non‐informative priors, 4 Markov Chain Monte Carlo (MCMC) chains, 5000 adaptations, and 20 000 iterations; analyses were conducted using the *BUGSnet* [14] package in *R* [15] software. We used comparators' Surface Under the Cumulative RAnking (SUCRA) values to rank efficacy (and safety); league tables—which are two‐dimensional—were used to present comparators' pairwise relative effect. We indicated FDA‐approved regimens in blue cells and significantly different effects in yellow highlights. For our safety‐related network, lower SUCRA values corresponded to more safety (i.e., less likely to discontinue due to AEs at 24 weeks of therapy).

## 3 Results

**FIGURE 1:**
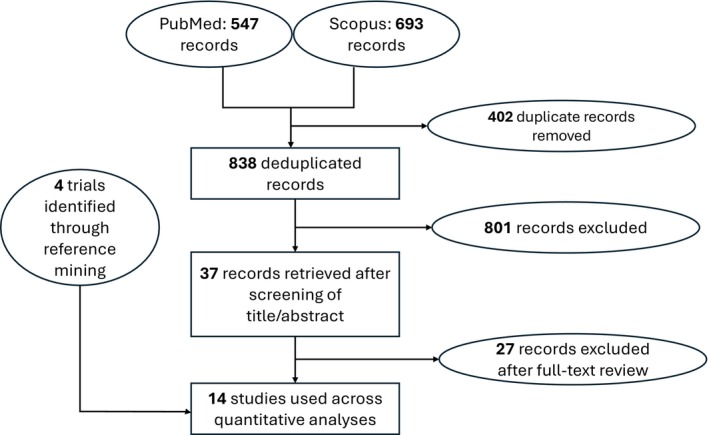
A schematic depicting the identification of studies whose data were used for quantitative analyses.

**FIGURE 2:**
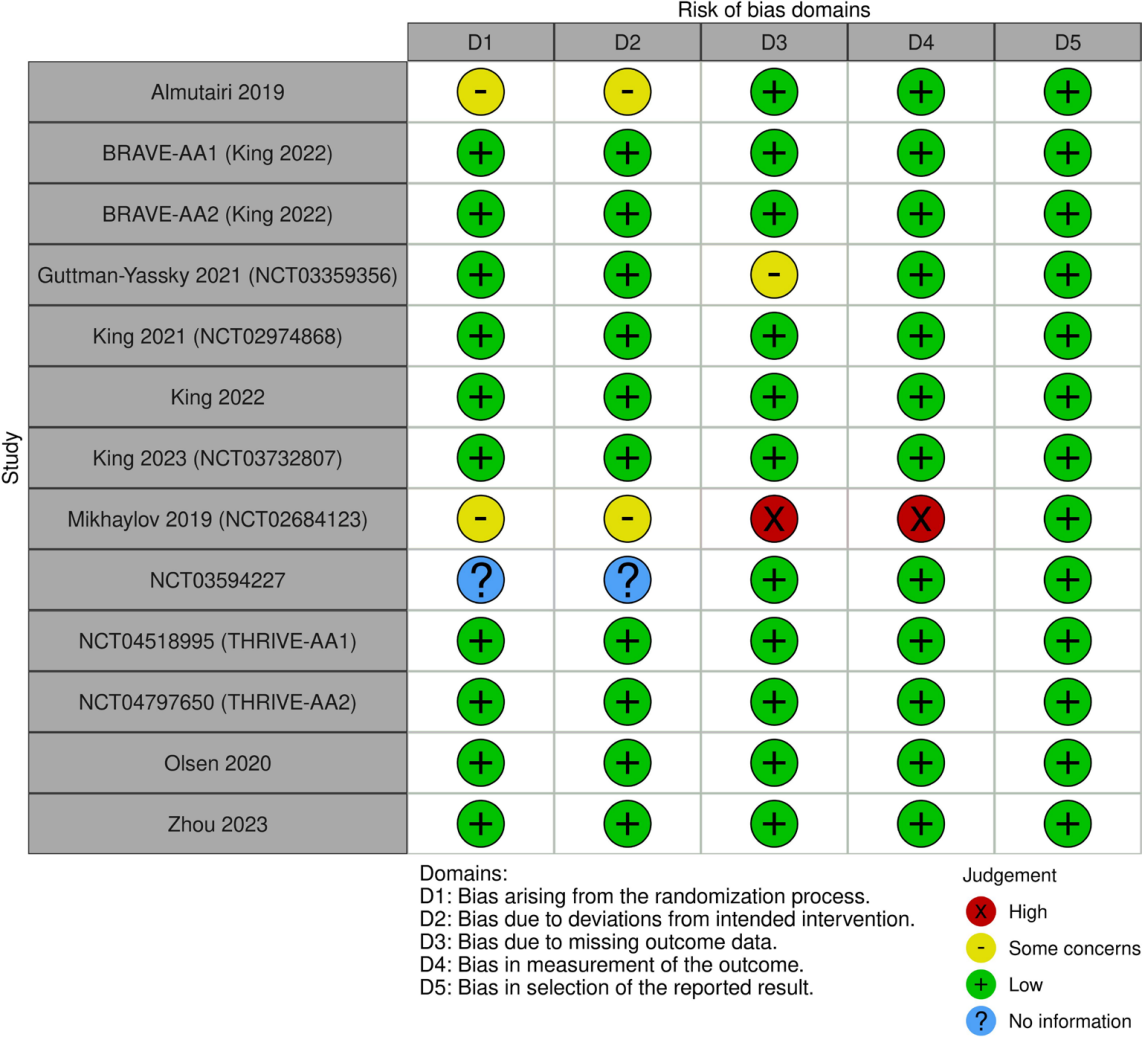
A qualitative summary of our evaluation of studies' evidence quality as per Cochrane Collaboration's Risk of Bias (Rob) tool.

Our search identified 14 trials that were used across the four networks for efficacy (Figure 1)—and the study characteristics thereof are summarized in Table 1. The study‐level evaluation of evidence quality, for each of the 14 trials, is presented in Figure 2. We deemed each efficacy network to be transitive after reviewing the study characteristics (Table 1). The four efficacy networks corresponded to the following outcomes, namely, percentage reduction in SALT score at 24 weeks from baseline (Figure S1), proportion of participants achieving a SALT score of 20 (or less) at 24 weeks from baseline (Figure S2), proportion of participants achieving a SALT score of 10 (or less) at 24 weeks from baseline (Figure S3) and proportion of participants achieving at least a 90% relative reduction in SALT at 24 weeks from baseline (Figure S4). The network structure for each outcome did not allow for node‐splitting analyses for inconsistency (i.e., no closed loops) [26].

**TABLE 1:** Characteristics of included studies.

Study	Intervention	Number of participants	Age (mean, standard deviation)	Sex (number of females, number of male females)
Almutairi 2019 [16]	Ruxolitinib 20 mg twice daily for 24 weeks	38	35.5, 13.8	17, 21
Almutairi 2019	Tofacitinib 5 mg twice daily for 24 weeks	37	47.4, 16.1	15, 22
BRAVE‐AA1 (King 2022) [17]	Baricitinib 2 mg once daily for 24 weeks	184	38, 12.8	109, 75
BRAVE‐AA1 (King 2022)	Baricitinib 4 mg once daily for 24 weeks	281	36.3, 13.3	165, 116
BRAVE‐AA1 (King 2022)	Placebo	189	37.4, 12.9	109, 80
BRAVE‐AA2 (King 2022) [17]	Baricitinib 2 mg once daily for 24 weeks	156	39, 13	103, 53
BRAVE‐AA2 (King 2022)	Baricitinib 4 mg once daily for 24 weeks	234	38, 12.7	144, 90
BRAVE‐AA2 (King 2022)	Placebo	156	37.1, 13	98, 58
Guttman‐Yassky 2021 (NCT03359356) [18]	Dupilumab 300 mg weekly for 24 weeks (subcutaneous)	40	41.6, 13.8	30, 10
Guttman‐Yassky 2021 (NCT03359356)	Placebo	20	46.5, 14.4	13, 7
King 2021 (NCT02974868) [19]	Brepocitinib 60 mg once daily for 4 weeks, then 30 mg once daily for 20 weeks	47	34, 11	32, 15
	Ritlecitinib 200 mg once daily for 4 weeks, then 50 mg once daily for 20 weeks	48	37, 13	37, 11
	Placebo	47	38, 14	29, 18
King 2022 [20]	Deuruxolitinib 12 mg twice daily for 24 weeks	37	35.8, 12.37	28, 9
	Deuruxolitinib 4 mg twice daily for 24 weeks	30	35.7, 11.01	22, 8
	Deuruxolitinib 8 mg twice daily for 24 weeks	38	37.3, 14.18	26, 12
	Placebo	44	37.8, 13.5	29, 15
King 2023 (NCT03732807) [21]	Ritlecitinib 10 mg once daily for 24 weeks	63	34.3, 13.9	43, 20
	Ritlecitinib 200 mg once daily for 4 weeks, then 30 mg once daily for 20 weeks	130	33.7, 13.8	85, 45
	Ritlecitinib 200 mg once daily for 4 weeks, then 50 mg once daily for 20 weeks	132	34.5, 15	81, 51
	Ritlecitinib 30 mg once daily for 24 weeks	132	33.7, 14.8	80, 52
	Ritlecitinib 50 mg once daily for 24 weeks	130	32.4, 13.4	71, 49
	Placebo	131	34, 15	86, 45
Mikhaylov 2019 (NCT02684123) [22]	Apremilast 30 mg twice daily for 24 weeks	12	37.1, 14.4	16, 4
	Placebo	9	44.15, 16.9	5, 5
NCT03594227	ATI‐501400 mg twice daily for 24 weeks	23	38.7, 12.99	17, 6
	ATI‐501600 mg twice daily for 24 weeks	23	40.4, 13.56	12, 11
	ATI‐501800 mg twice daily for 24 weeks	22	40.5, 12.44	13, 9
	Placebo	19	41.8, 16.01	14, 5
NCT04518995 (THRIVE‐AA1) [23]	Deuruxolitinib 12 mg twice daily for 24 weeks	215	38.2, 12.80	131, 84
	Deuruxolitinib 8 mg twice daily for 24 weeks	351	38.9, 13.32	217, 134
	Placebo	128	38.7, 13.81	89, 51
NCT04797650 (THRIVE‐AA2)	Deuruxolitinib 12 mg twice daily for 24 weeks	129	39.7, 12.90	84, 45
	Deuruxolitinib 8 mg twice daily for 24 weeks	258	38.4, 12.30	177, 81
	Placebo	119	39.7, 12.49	88, 42
Olsen 2020 [24]	1.5% Ruxolitinib cream once daily for 24 weeks	33	44.3, 12.5	24, 15
	Vehicle	33	42.3, 12.5	27, 12
Zhou 2023 [25]	Ivarmacitinib 2 mg once daily for 24 weeks	18	36, 10.4	11, 12
	Ivarmacitinib 4 mg once daily for 24 weeks	22	33.3, 12.2	14, 9
	Ivarmacitinib 8 mg once daily for 24 weeks	20	37.9, 13.7	15, 9
	Placebo	19	34.7, 9.4	12, 12

Excluding placebo/vehicle, 23 active comparators were identified across the four efficacy networks. The 23 comparators corresponded to the following regimens: (1) “brepocitinib 60 mg once daily for 4 weeks then 30 mg once daily for 20 weeks,” (2) “deuruxolitinib 12 mg twice daily for 24 weeks,” (3) “ritlecitinib 200 mg once daily for 4 weeks then 50 mg once daily for 20 weeks,” (4) “baricitinib 4 mg once daily for 24 weeks,” (5) “baricitinib 2 mg once daily for 24 weeks,” (6) “1.5% ruxolitinib cream once daily for 24 weeks,” (7) “deuruxolitinib 8 mg twice daily for 24 weeks,” (8) “ivarmacitinib 4 mg once daily for 24 weeks,” (9) “ivarmacitinib 8 mg once daily for 24 weeks,” (10) “dupilumab 300 mg weekly for 24 weeks (subcutaneous),” (11) “ivarmacitinib 2 mg once daily for 24 weeks,” (12) “deuruxolitinib 4 mg twice daily for 24 weeks,” (13) “apremilast 30 mg twice daily for 24 weeks,” (14) “ATI‐501 400 mg twice daily for 24 weeks,” (15) “ATI‐501 600 mg twice daily for 24 weeks,” (16) “ATI‐501 800 mg twice daily for 24 weeks,” (17) “ritlecitinib 10 mg once daily for 24 weeks,” (18) “ritlecitinib 200 mg once daily for 4 weeks then 30 mg once daily for 20 weeks,” (19) “ritlecitinib 30 mg once daily for 24 weeks,” (20) “ritlecitinib 30mg once daily for 24 weeks,” (21) “ritlecitinib 50 mg once daily for 24 weeks,” (22) “ruxolitinib 20 mg twice daily for 24 weeks”, and (23) “tofacitinib 5 mg twice daily for 24 weeks.”

**FIGURE 3:**
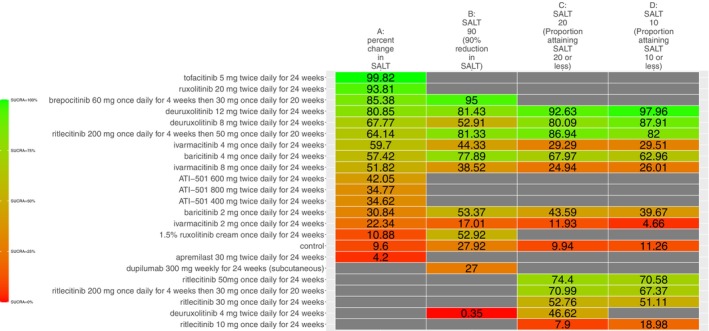
A kilim plot of regimens' Surface Under the Cumulative RAnking (SUCRA) values. The four efficacy outcomes are depicted in this kilim plot. The closer a regimen's SUCRA value is to green, the more efficacious it is; the closer a regimen's SUCRA value is to red, the less efficacious it is. Regimens are ordered according to the decreasing SUCRA value for “percentage reduction in SALT score at 24 weeks from baseline”. The order of regimens' SUCRA values as per the three other outcomes were congruent with that of for “percentage reduction in SALT score at 24 weeks from baseline”—as evidenced by similar “color gradients” for each outcome. SALT = Severity of Alopecia Tool. The regimens highlighted in blue correspond to agents approved by the United States Food and Drug Administration for the treatment of alopecia areata.

**FIGURE 4:**
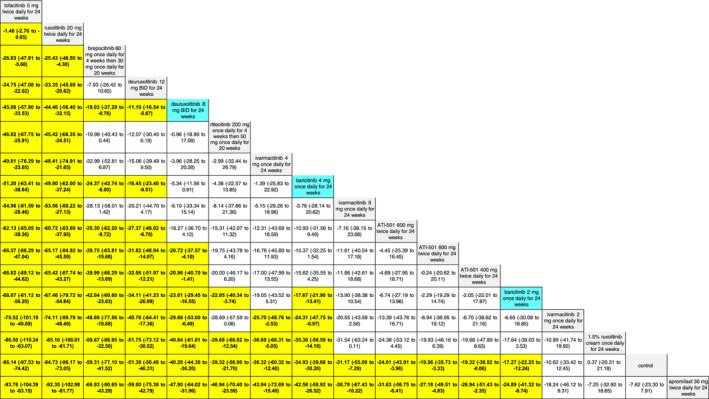
League table for “percentage reduction in a Severity of Alopecia Tool (SALT) score at 24 weeks from baseline”. Each cell represents regimens' pairwise relative effect in terms of the mean difference (MD) and 95% credible interval (CI). The yellow‐highlighted cells correspond to regimens whose effects are significantly different (i.e., *p* < 0.05) from each other; the regimens in blue cells correspond to the regimens approved by the United States Food and Drug Administration for treatment of alopecia areata.

**FIGURE 5:**
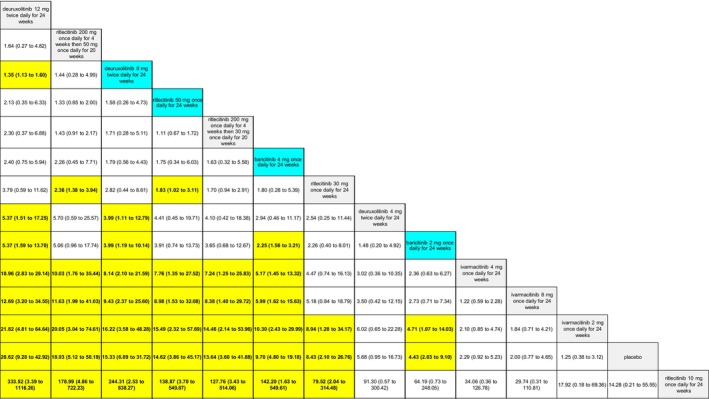
League table for “proportion of participants achieving a Severity of Alopecia Tool (SALT) score of 20 (or less) at 24 weeks from baseline”. Each cell represents regimens' pairwise relative effect in terms of the odds ratio (OR) and 95% credible interval (CI). The yellow‐highlighted cells correspond to regimens whose effects are significantly different (i.e., *p* < 0.05) from each other; the regimens in blue cells correspond to the regimens approved by the United States Food and Drug Administration for treatment of alopecia areata.

**FIGURE 6:**
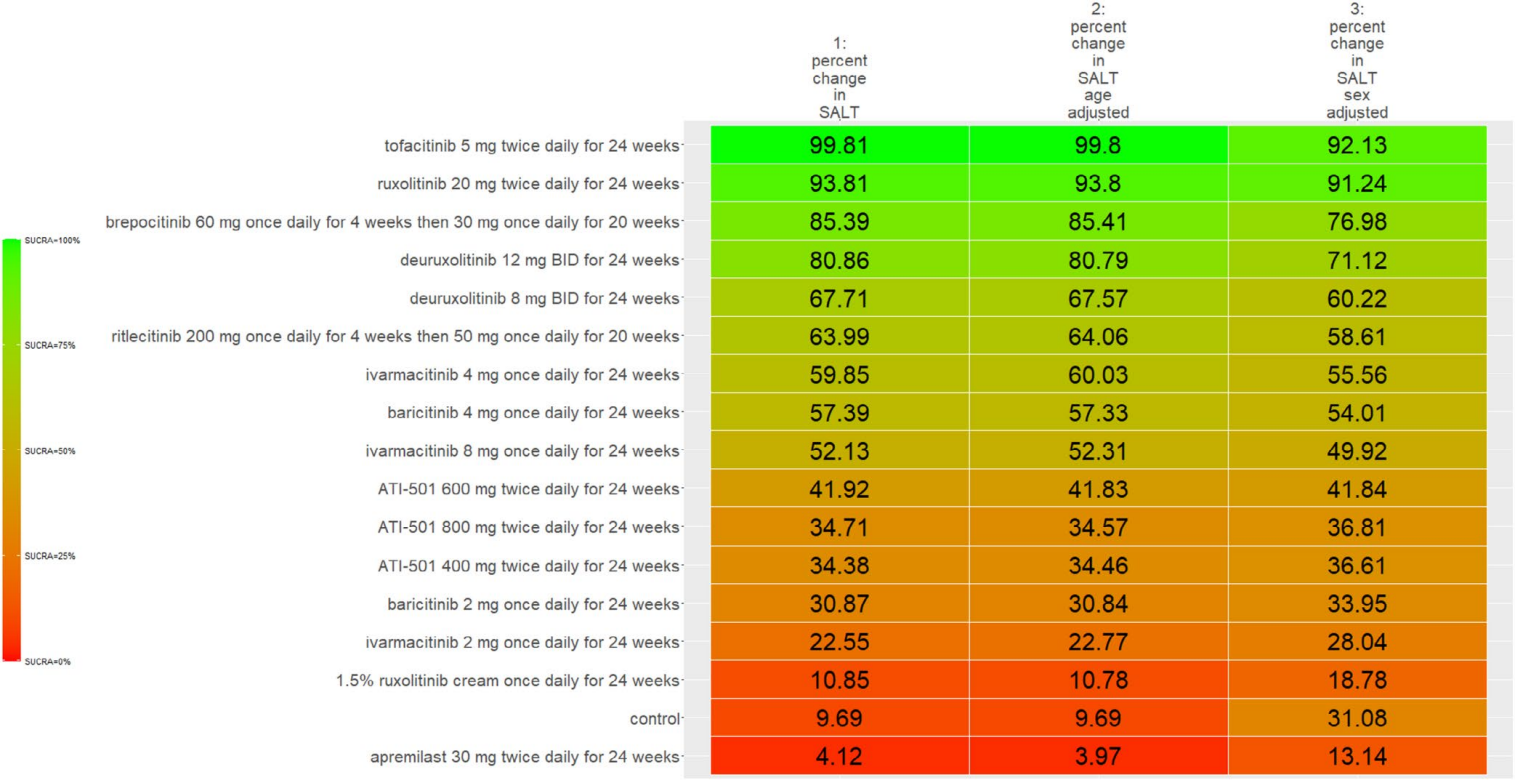
A kilim plot of the regimens' surface under the cumulative ranking (SUCRA) values from base (i.e., main) analyses and sensitivity analyses for “proportion of participants achieving a Severity of Alopecia Tool (SALT) score of 20 (or less) at 24 weeks from baseline”. For sensitivity analyses, two separate network meta‐regression analyses were conducted, where one (ecologically) adjusted for variation due to age (i.e., age‐adjusted) and the other adjusted for variation due to sex (i.e., sex‐adjusted). The closer a regimen's SUCRA value is to green, the more efficacious it is; the closer a regimen's SUCRA value is to red, the less efficacious it is. SALT = Severity of Alopecia Tool.

**FIGURE 7:**
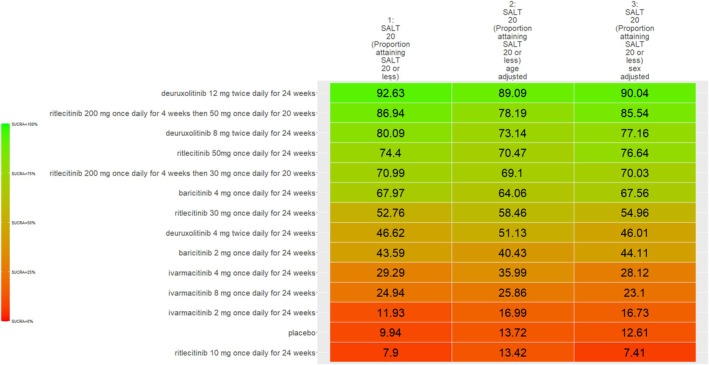
A kilim plot of the regimens' surface under the cumulative ranking (SUCRA) values from base (i.e., main) analyses and sensitivity analyses for “percentage reduction in a Severity of Alopecia Tool (SALT) score at 24 weeks from baseline”. For sensitivity analyses, two separate network meta‐regression analyses were conducted, where one (ecologically) adjusted for variation due to age (i.e., age‐adjusted) and the other adjusted for variation due to sex (i.e., sex‐adjusted). The closer a regimen's SUCRA value is to green, the more efficacious it is; the closer a regimen's SUCRA value is to red, the less efficacious it is. SALT = Severity of Alopecia Tool.

The SUCRA values of active comparators, for each of the 4 efficacy networks, are presented in a kilim plot (Figure 3). Comparators' pairwise relative effects—as per “percentage reduction in SALT score at 24 weeks from baseline”—are presented in the league table in Figure 4. The league table in Figure 5 details comparators' pairwise relative effects in terms of “proportion of participants achieving a SALT score of 20 (or less) at 24 weeks from baseline”. League tables for the other two efficacy endpoints are presented in the Figures S5 and S6. Mean difference (MD) was the point estimate in the league table for the network pertaining to “percentage reduction in SALT score at 24 weeks from baseline”; for the other three networks, the odds ratio (OR) was the point estimate used. The 95% credible intervals (95% CI) for all point estimates were reported in parentheses (Figures 4 and 5 and Figures S5 and S6). Results from our sensitivity analyses are presented in Figures 6 and 7; we conducted for just “percentage reduction in SALT score at 24 weeks from baseline” and “proportion of participants achieving a SALT score of 20 (or less) at 24 weeks from baseline” as these are main endpoints in AA efficacy literature.

The safety outcome (i.e., discontinuation due to AEs at 24 weeks) was a secondary outcome and data from safety NMA came from the 14 trials. The network plot, and league table, for “proportion of patients who discontinued therapy due to AEs at 24 weeks from baseline” are detailed in Figure S7 and Table S1 of the Appendix S1.

## 4 Discussion

The relative efficacy of the JAKIs in the management of AA is shown in Figure 3. The current NMA is the first to provide quantitative evidence on the relative efficacy of various AA monotherapies, including the FDA‐approved JAKIs (baricitinib [FDA approval, 2022] 2 mg/day increase to 4 mg/day if dose is not adequate, ritlecitinib [FDA approval, 2023] 50 mg/day, and deuruxolitinib [FDA approval, 2024] 8 mg twice daily). We looked at the proportion of individuals achieving a SALT score of 20 (or less) at week 24 (Figure 5). There was no significant difference in efficacy between deuruxolitinib 8 mg twice daily, ritlecitinib 50 mg once daily, and baricitinib 4 mg per day. However, deuruxolitinib 8 mg twice daily may be more effective than baricitinib 2 mg per day.

Using data from RCTs, we determined the relative efficacy of specific monotherapies (JAKIs, apremilast, and dupilumab) through Bayesian NMAs. The current work has some strengths. To avoid “time varying confounding” we analyzed outcomes that were measured at a specific timepoint (i.e., only 24 weeks), unlike some NMAs such as the one by Wei et al. [8]—where outcome data, per network, was amalgamated from different timepoints including 24, 36, and 12 weeks. Our findings have some congruence with NMAs that amalgamated non‐randomized evidence with RCTs; in this NMA we used only RCTs. By restricting our analyses to only data from randomized trials, the RoB across our networks is lower than if observational studies had been included.

The current NMA produced comparative evidence using 6‐month outcome data. As empirical evidence regarding the effects of JAKIs on AA expands, more comparative analyses can be done for longer term efficacy (e.g., 1‐year efficacy). Also, future studies can more critically investigate relative safety by using additional safety outcome measures to better understand the regimens' safety profile. The SUCRA values and relative effects that were estimated through our NMAs exhibited a dose‐dependent relationship. We found, for SALT_20_, “deuruxolitinib 12 mg twice daily for 24 weeks” was more efficacious than “deuruxolitinib 8 mg twice daily for 24 weeks” (OR = 1.35, 95% CI = 1.13, 1.6, *p* < 0.05) (Figure 5)—and “deuruxolitinib 8 mg twice daily for 24 weeks” was more efficacious than “deuruxolitinib 4 mg twice daily for 24 weeks” (OR = 3.99, 95% CI = 1.11, 12.79, *p* < 0.05) (Figure 5). Similarly, “baricitinib 4 mg once daily for 24 weeks” is more efficacious than “baricitinib 2 mg once daily for 24 weeks” insofar as SALT_20_ (OR = 2.25, 95% CI = 1.56, 3.21, *p* < 0.05). This dose‐dependency was also observed with other outcome measures. For example, “baricitinib 4 mg once daily for 24 weeks” is more efficacious than “baricitinib 2 mg once daily for 24 weeks” for SALT_10_ (OR = 2.5, 95% CI = 1.64, 3.77, *p* < 0.05) (Figure S5).

We chose “proportion of participants achieving a SALT score of 20 (or less)” as one of our endpoints because it has been deemed a clinically relevant outcome for this condition [12]. For percentage of patients who achieved at least 90% reduction in SALT score at 24 weeks, there is a notable degree of congruency in comparators' SUCRAs values between the former [3] and updated (i.e., current) NMA studies [5]; for instance, “brepocitinib 60 mg once daily for 4 weeks then 30 mg once daily for 20 weeks” was ranked most efficacious in both the former (SUCRA = 92%) [5] as well as the present (SUCRA = 95%) studies; similarly, “deuruxolitinib 4 mg twice daily for 24 weeks” was ranked the least efficacious regimen in both in terms of the same outcome (i.e., percentage of patients who achieved at least 90% reduction in SALT score at 24 weeks).

Across the four networks (i.e., the networks for efficacy), we investigated the relative efficacy of 23 active comparators (Figure 3). Since the previous NMA study [5], the current one included data from 6 new RCTs—two of which compared agents that the previous NMA study did not (i.e., ivarmacitinib 2 mg once daily for 24 weeks, ivarmacitinib 4 mg once daily for 24 weeks, ivarmacitinib 8 mg once daily for 24 weeks, ATI‐501400 mg twice daily for 24 weeks, ATI‐501600 mg twice daily for 24 weeks and ATI‐501 800 mg twice daily for 24 weeks).

In addition to producing new findings, results from the present work also resonate with those of previous studies. For instance, Yan et al. [27] also determined the comparative efficacy of JAKIs; however, the current study in addition to using SALT_20_ (i.e., number of subjects who achieved a SALT score of 20 or less), also examined higher cutoff values like SALT_90_ (proportion of participants achieving at least a 90% improvement) and SALT_10_ (proportion of participants who achieved a SALT score of 10 or less). Yan et al. [27] did not investigate SALT_10_ nor SALT_90_. The SUCRA rank order for SALT_20_ in the present NMA study (Figure 3) and Yan et al.'s [27] are highly congruent.

For the discontinuation due to any AE at 24 weeks, we found differences in safety as evidenced by the different SUCRA values (Table S1), albeit the differences were not statistically significant (i.e., *p* ≥ 0.05) as per the league table (data not shown).

A limitation of our work is the arguably small sample size of studies per network (i.e., per outcome measure); given that sample size and credible intervals are inversely related, some of the wide 95% CIs of our pairwise comparisons can be attributed to sample size. We conducted sensitivity analyses despite the arguably small size of the included studies. The congruent color gradient of the Kilim plots for sensitivity analyses (Figures 6 and 7) supports that our base analyses were robust: regimens' similar color gradient corresponds to there being a similar rank order of efficacy for the age‐adjusted and sex‐adjusted analyses. However, point estimates for the pairwise relative effects from the network meta‐regressions had—by and large—much wider credible intervals (data not shown). This would be due to the network meta‐regression being less statistically powered than the base analyses. Hence, a major research implication of the current study is that our findings justify the conduct of similar but larger‐sized (i.e., well statistically‐powered) RCTs—to consequently provide empirical evidence that would, in turn, allow for a meaningful repertoire of analyses including analyses of “effect modification”—also known as “moderation” in epidemiology. Hence, future works would allow “heterogeneity of effects,” for AA monotherapies, to be well studied. For example, studies on the heterogeneity of effects can include investigating the effect of age (or age groups), sex, or even duration of diagnoses. More empirical evidence would also allow for better analyses for these drugs' safety profiles and even better understand predictors of treatment discontinuation. While our work provided evidence on deuruxolitinib's efficacy, the agent is not commercially available (March 2025).

## 5 Conclusion

Our work makes a case for the conduct of actual head‐to‐head multi‐arm trials. The results can guide clinical decision‐making and potentially update clinical practice guidelines.

## Author Contributions

Conception of the manuscript was done by A.K.G. and M.T. The manuscript was drafted by M.A.B. and A.K.G., substantively edited, and revised by A.K.G., M.A.B., P.M., V.P., and M.T.

## Ethics Statement

Approval from an ethics board was not required as there was no direct involvement with human participants.

## Conflicts of Interest

A.K.G., M.A.B., P.M., and M.T. have no conflicts of interest to declare. V.P. has received grants from AbbVie, Bausch Health, Celgene, Eli Lilly, Incyte, Janssen, LEO Pharma, L'Oréal, Novartis, Organon, Pfizer, Sandoz, and Sanofi, received payment or honoraria for speaking engagement from Sanofi China, participated on an advisory board for LEO Pharma, Novartis, Sanofi, and Union Therapeutics, and received equipment donation from L'Oréal. V.P. declares that the interests do not affect the objectivity or integrity of this article.

## Supporting information


Appendix S1:

## Data Availability

Data available on request from the authors.
