# Supplementary material for: The Relative Efficacy and Safety of Monotherapies for Alopecia Areata: A Network Meta‐Analysis Study
Source: J Cosmet Dermatol. 2025 Apr 15;24(4):e70185. doi: 10.1111/jocd.70185 (PMC11998889; doi:10.1111/jocd.70185)
Supplement: Supplementary file 1 — Appendix S1: [file JOCD-24-e70185-s001.pdf]

Supplementary Figure 1. Network plot for percentage reduction in SALT score at 24 weeks from baseline

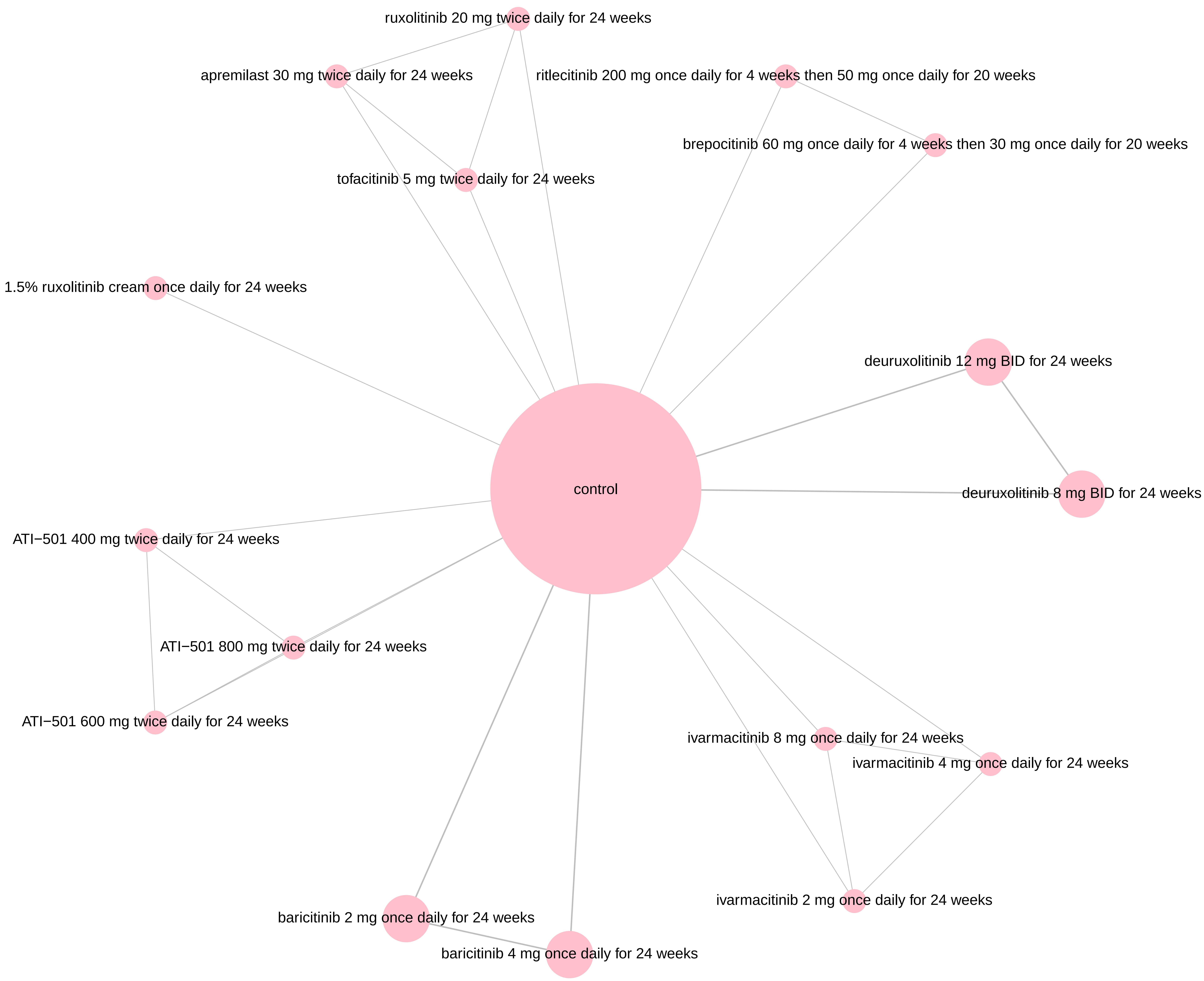

Supplementary Figure 2. Network plot for proportion of participants achieving a SALT score of 20 (or less) at 24 weeks from baseline 3

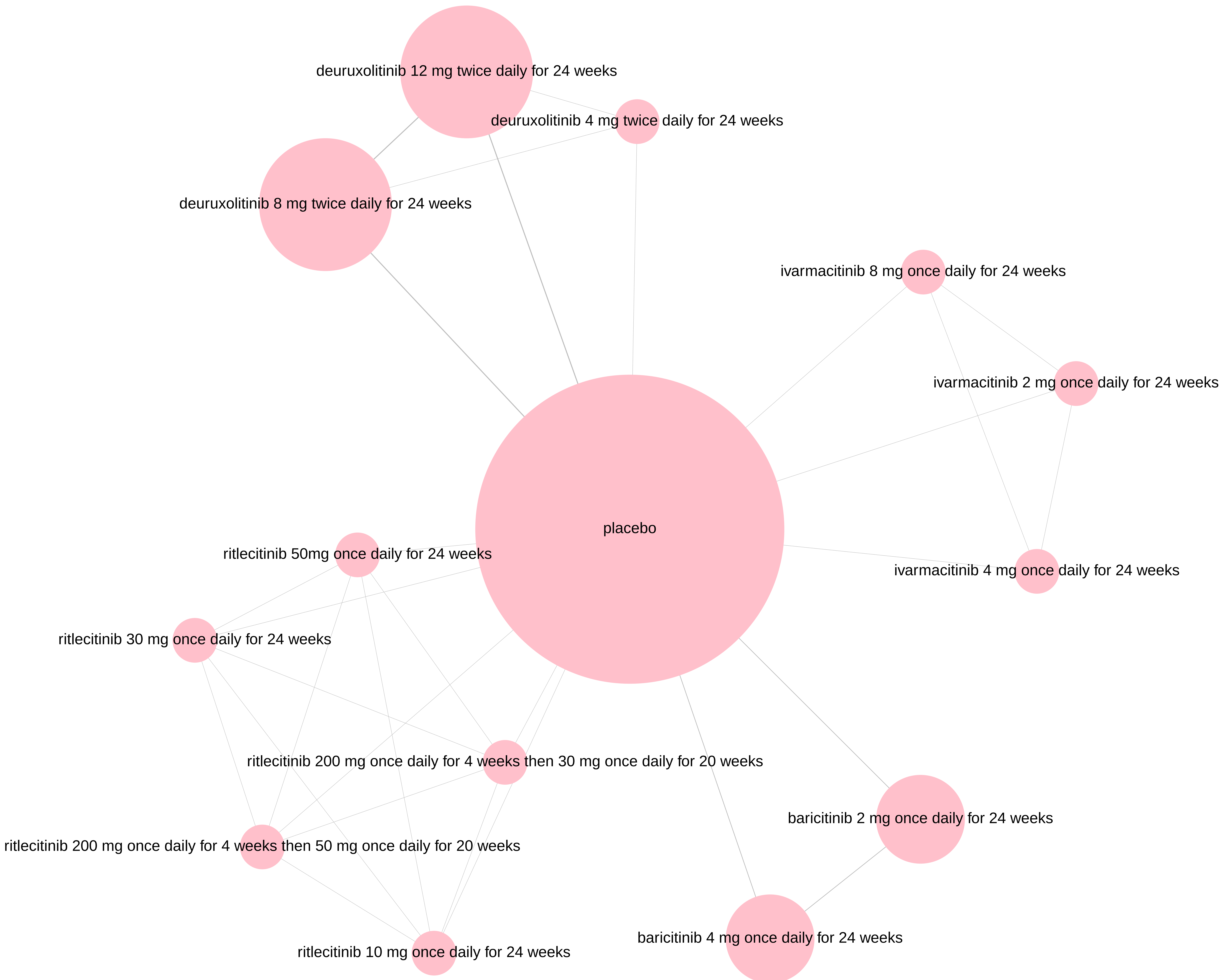

Supplementary Figure 3. Network plot for proportion of participants achieving a SALT score of 10 (or less) at 24 weeks from baseline

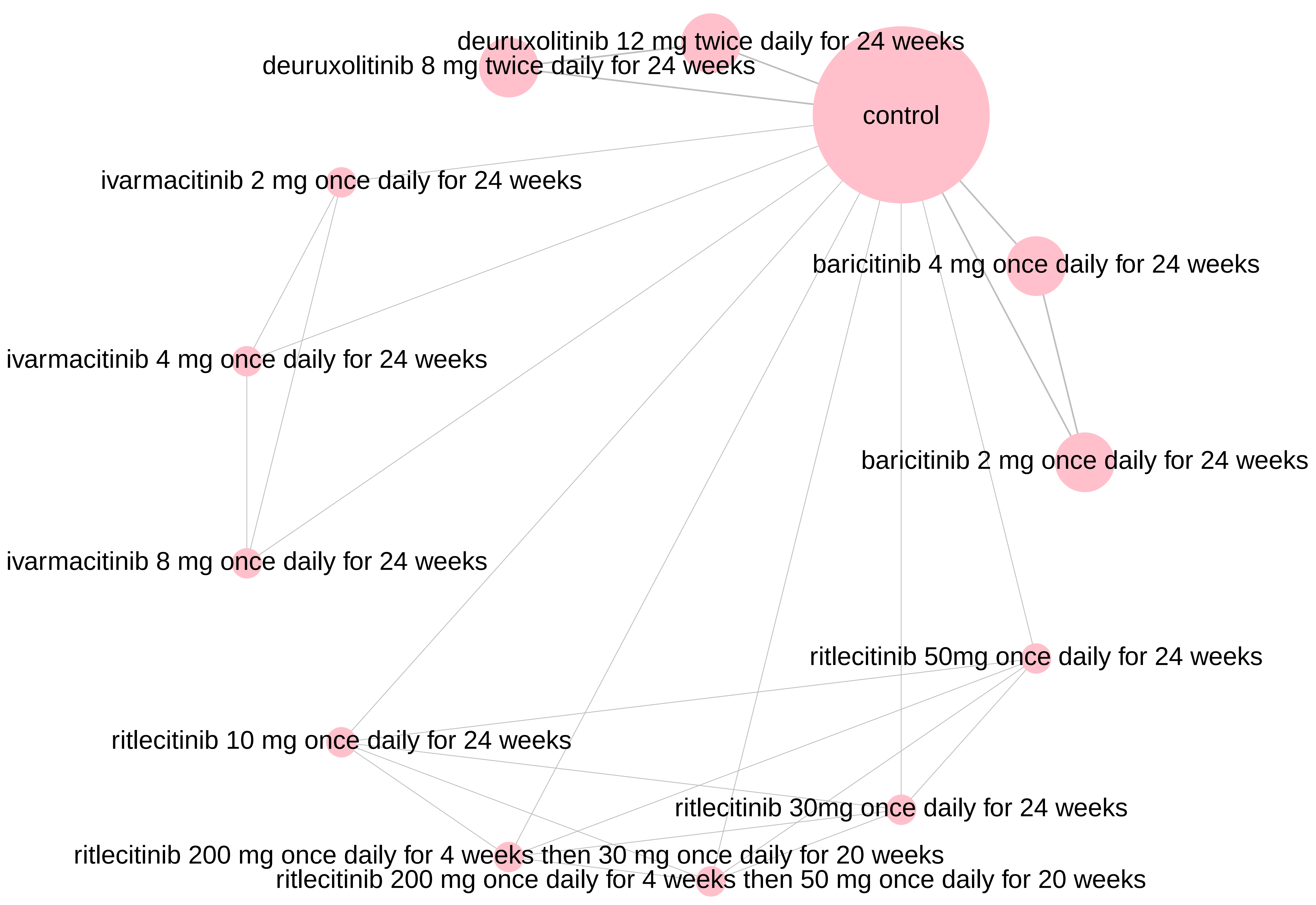

Supplementary Figure 4. Network plot for proportion of participants achieving at least a 90% relative reduction in SALT at 24 weeks from baseline

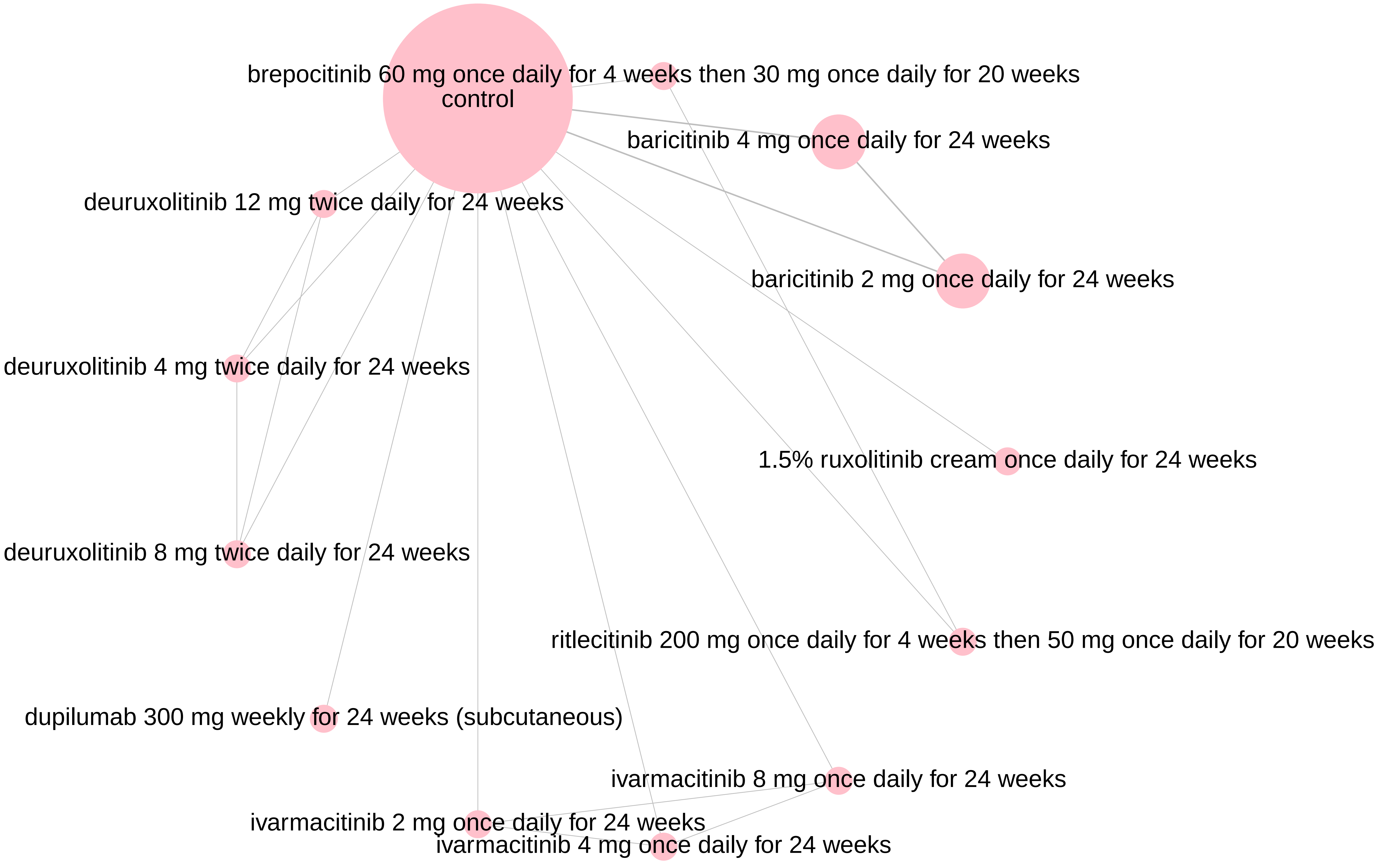

Supplementary Figure 5. Relative effects for the proportion of participants achieving a SALT score of 10 (or less) at 24 weeks

**deuruxolitinib 12 mg twice daily for 24 weeks**												
**1.41 (1.12 to 1.74)**	**deuruxolitinib 8 mg twice daily for 24 weeks**											
6.34 (0.46 to 30.34)	4.53 (0.33 to 21.66)	**ritlecitinib 200 mg once daily for 4 weeks then 50 mg once daily for 20 weeks**										
8.25 (0.59 to 39.89)	5.90 (0.42 to 28.32)	1.33 (0.85 to 1.98)	**ritlecitinib 50 mg once daily for 24 weeks**									
8.91 (0.63 to 42.61)	6.36 (0.45 to 30.49)	1.43 (0.91 to 2.17)	1.11 (0.67 to 1.73)	**ritlecitinib 200 mg once daily for 4 weeks then 30 mg once daily for 20 weeks**								
**10.97 (1.39 to 49.08)**	7.84 (1.00 to 35.05)	2.67 (0.48 to 10.28)	2.07 (0.37 to 8.01)	1.93 (0.34 to 7.46)	**baricitinib 4 mg once daily for 24 weeks**							
**14.69 (1.02 to 70.77)**	10.50 (0.73 to 50.62)	**2.36 (1.38 to 3.93)**	1.83 (1.02 to 3.11)	1.70 (0.94 to 2.93)	1.62 (0.22 to 4.96)	**ritlecitinib 30mg once daily for 24 weeks**						
**27.25 (3.30 to 123.87)**	**19.48 (2.36 to 88.75)**	**6.64 (1.13 to 25.94)**	5.15 (0.87 to 20.29)	4.79 (0.80 to 18.84)	**2.50 (1.64 to 3.77)**	2.97 (0.47 to 11.74)	**baricitinib 2 mg once daily for 24 weeks**					
**47.48 (5.34 to 226.36)**	**33.91 (3.83 to 161.39)**	**11.86 (1.60 to 47.92)**	**9.18 (1.22 to 37.26)**	**8.56 (1.14 to 34.88)**	**5.05 (1.19 to 13.99)**	5.30 (0.68 to 21.77)	2.09 (0.46 to 5.96)	**ivarmacitinib 4 mg once daily for 24 weeks**				
**54.16 (5.94 to 256.87)**	**38.71 (4.26 to 184.53)**	**13.55 (1.77 to 55.04)**	**10.51 (1.35 to 43.11)**	**9.79 (1.26 to 39.94)**	**5.75 (1.32 to 16.31)**	6.06 (0.75 to 24.90)	2.38 (0.51 to 6.92)	1.23 (0.51 to 2.56)	**ivarmacitinib 8 mg once daily for 24 weeks**			
**109.98 (3.78 to 626.61)**	**78.47 (2.69 to 445.53)**	**17.72 (3.21 to 72.33)**	**13.76 (2.43 to 56.44)**	**12.79 (2.25 to 52.17)**	12.24 (0.76 to 58.19)	**7.92 (1.31 to 32.91)**	5.08 (0.30 to 24.61)	3.16 (0.16 to 15.84)	2.83 (0.14 to 14.10)	**ritlecitinib 10 mg once daily for 24 weeks**		
**86.73 (16.07 to 400.21)**	**62.00 (11.43 to 287.01)**	**21.21 (5.07 to 77.83)**	**16.45 (3.87 to 60.27)**	**15.31 (3.59 to 56.46)**	**9.07 (4.34 to 18.31)**	**9.48 (2.10 to 35.34)**	**3.76 (1.63 to 7.94)**	2.33 (0.79 to 5.82)	2.07 (0.67 to 5.26)	2.31 (0.16 to 10.16)	**control**	
**196.54 (14.41 to 1023.02)**	**140.42 (10.30 to 730.01)**	**49.10 (4.29 to 232.96)**	**38.11 (3.29 to 181.23)**	**35.40 (3.05 to 167.89)**	**20.77 (3.12 to 78.85)**	**21.94 (1.83 to 104.32)**	**8.61 (1.22 to 33.57)**	**4.45 (1.11 to 14.69)**	3.96 (0.96 to 13.06)	5.35 (0.19 to 28.30)	2.29 (0.44 to 7.93)	**ivarmacitinib 2 mg once daily for 24 weeks**

Supplementary Figure 6. Relative effects for the proportion of participants achieving at least a 90% relative reduction in SALT at 24 weeks from baseline

**brepocitinib 60 mg once daily for 4 weeks then 30 mg once daily for 20 weeks**												
15.55 (0.18 to 80.70)	**deuruxolitinib 12 mg twice daily for 24 weeks**											
2.13 (0.93 to 4.46)	2.03 (0.02 to 11.46)	**ritlecitinib 200 mg once daily for 4 weeks then 50 mg once daily for 20 weeks**										
14.04 (0.45 to 74.23)	2.14 (0.26 to 9.64)	7.31 (0.21 to 39.21)	**baricitinib 4 mg once daily for 24 weeks**									
87.63 (0.17 to 478.47)	12.64 (0.08 to 79.52)	44.23 (0.09 to 244.73)	7.15 (0.08 to 39.43)	**1.5% ruxolitinib cream once daily for 24 weeks**								
**39.77 (1.21 to 212.57)**	6.12 (0.70 to 27.75)	20.69 (0.58 to 111.66)	**2.89 (1.75 to 4.65)**	11.68 (0.07 to 34.64)	**baricitinib 2 mg once daily for 24 weeks**							
59.00 (0.53 to 312.85)	**4.01 (1.24 to 11.37)**	30.41 (0.26 to 165.22)	4.28 (0.30 to 17.21)	17.97 (0.04 to 49.31)	1.56 (0.10 to 6.38)	**deuruxolitinib 8 mg twice daily for 24 weeks**						
**66.69 (1.50 to 373.16)**	10.26 (0.83 to 49.85)	34.57 (0.71 to 195.46)	5.50 (0.97 to 17.32)	22.55 (0.09 to 61.11)	2.00 (0.33 to 6.50)	3.33 (0.19 to 16.90)	**ivarmacitinib 4 mg once daily for 24 weeks**					
**83.85 (1.75 to 466.33)**	12.59 (0.97 to 62.85)	42.90 (0.83 to 241.46)	**6.81 (1.12 to 22.30)**	28.02 (0.10 to 76.08)	2.49 (0.38 to 8.42)	4.07 (0.23 to 21.12)	1.43 (0.40 to 3.77)	**ivarmacitinib 8 mg once daily for 24 weeks**				
**94.98 (3.82 to 503.13)**	**13.98 (2.30 to 61.67)**	**49.50 (1.79 to 263.14)**	**7.64 (3.60 to 15.57)**	32.16 (0.20 to 84.72)	**2.78 (1.14 to 6.06)**	4.53 (0.48 to 20.61)	2.07 (0.52 to 6.15)	1.73 (0.39 to 5.25)	**control**			
1130.97 (0.75 to 4690.37)	191.65 (0.36 to 918.67)	600.18 (0.37 to 2416.77)	144.30 (0.35 to 546.66)	406.02 (0.06 to 712.60)	52.25 (0.12 to 198.36)	60.60 (0.10 to 291.22)	33.57 (0.07 to 149.20)	27.32 (0.06 to 127.45)	17.51 (0.05 to 72.17)	**dupilumab 300 mg weekly for 24 weeks (subcutaneous)**		
**336.80 (4.07 to 1836.08)**	**50.80 (2.17 to 288.58)**	**179.00 (1.95 to 946.79)**	**28.09 (2.48 to 131.53)**	118.25 (0.26 to 290.47)	10.23 (0.85 to 48.85)	16.60 (0.53 to 94.40)	5.86 (0.84 to 24.70)	4.91 (0.65 to 20.99)	3.66 (0.41 to 15.93)	18.67 (0.02 to 68.03)	**ivarmacitinib 2 mg once daily for 24 weeks**	
												**deuruxolitinib 4 mg twice daily for 24 weeks**

Note: the bottom row is blank because the point estimates in last row (i.e., yellow cells that are blank) were so large and statistically signfiicant (p<0.05) with higly wide 95% credible intervals.

Supplementary Figure 7. Network plot for safety-related outcome
(i.e., the proportion of participants who discontinued therapy due to adverse events at 24 weeks from baseline).

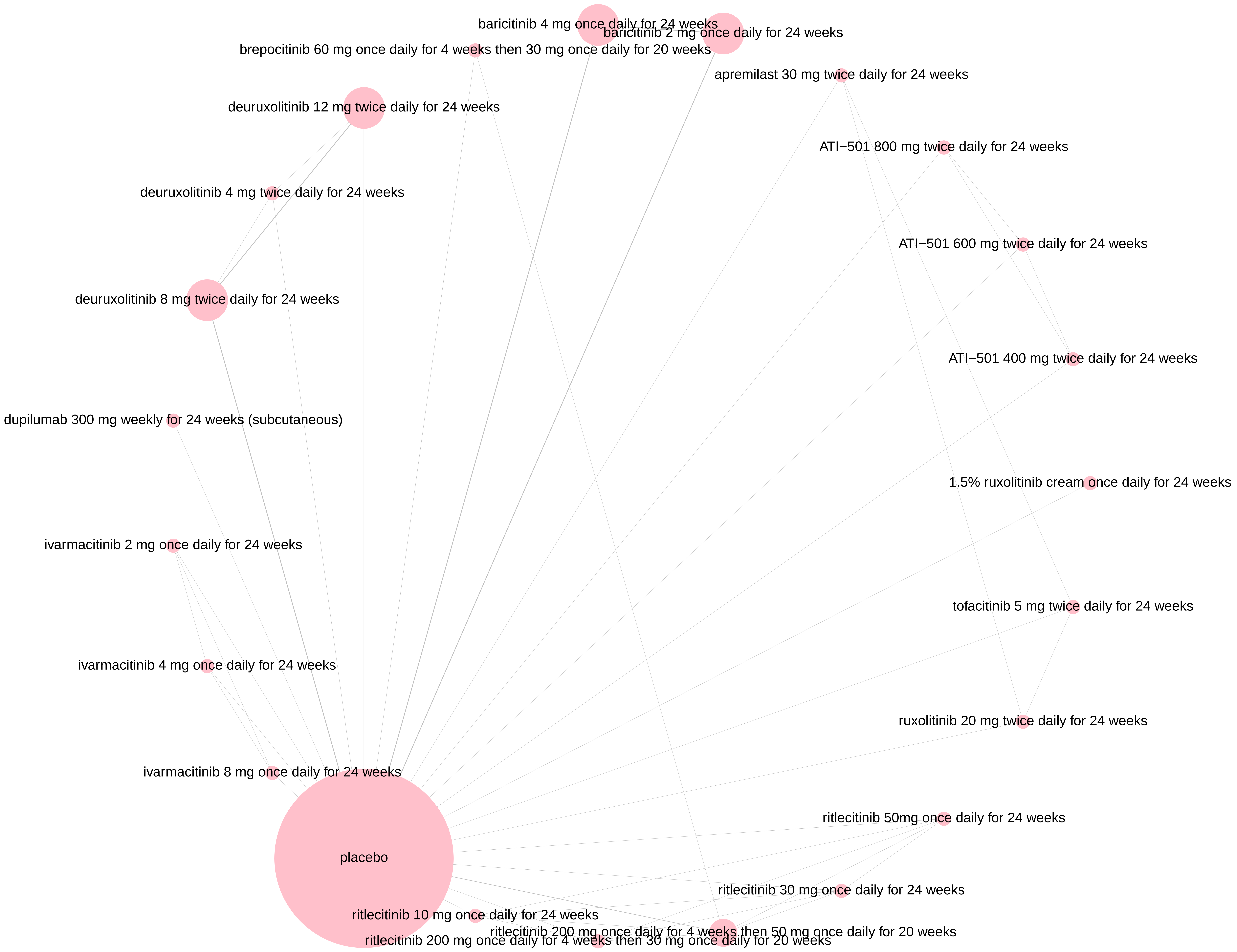

Supplementary Table 1. SUCRA for the safety-related outcome (i.e., the proportion of participants who discontinued therapy due to adverse events at 24 weeks from baseline).

Regimen	SUCRA (%)
ritlecitinib 200 mg once daily for 4 weeks then 30 mg once daily for 20 weeks	82.06
ivarmacitinib 4 mg once daily for 24 weeks	81.65
deuruxolitinib 4 mg twice daily for 24 weeks	81.45
ruxolitinib 20 mg twice daily for 24 weeks	81.28
1.5% ruxolitinib cream once daily for 24 weeks	81.2
tofacitinib 5 mg twice daily for 24 weeks	81.14
ATI-501 400 mg twice daily for 24 weeks	80.23
ATI-501 600 mg twice daily for 24 weeks	80.23
ATI-501 800 mg twice daily for 24 weeks	80.22
ivarmacitinib 2 mg once daily for 24 weeks	46.1
ivarmacitinib 8 mg once daily for 24 weeks	45.55
ritlecitinib 50 mg once daily for 24 weeks	41.45
dupilumab 300 mg weekly for 24 weeks (subcutaneous)	34.21
ritlecitinib 200 mg once daily for 4 weeks then 50 mg once daily for 20 weeks	33.96
deuruxolitinib 12 mg twice daily for 24 weeks	33.56
baricitinib 2 mg once daily for 24 weeks	29.19
deuruxolitinib 8 mg twice daily for 24 weeks	26.61
ritlecitinib 10 mg once daily for 24 weeks	26.27
ritlecitinib 30 mg once daily for 24 weeks	24.36
baricitinib 4 mg once daily for 24 weeks	23.68
brepocitinib 60 mg once daily for 4 weeks then 30 mg once daily for 20 weeks	22.44
placebo	21.25
apremilast 30 mg twice daily for 24 weeks	11.91

Abbreviations: SUCRA = Surface Under the Cumulative RAnking
